# Multi-dimensional epidemiology and informatics data on COVID-19 wave at the end of zero COVID policy in China

**DOI:** 10.3389/fpubh.2024.1442728

**Published:** 2024-08-19

**Authors:** Xin-sheng Yu, Shaoying Tan, Wanting Tang, Fang-fang Zhao, Jie Ji, Jianwei Lin, Han-jie He, Youxin Gu, Jia-Jian Liang, Meng Wang, Yequn Chen, Jiancheng Yang, Longxu Xie, Qian Wang, Mengyu Liu, Yang He, Lan Chen, Ya Xing Wang, Zhaoxiong Wu, Gang Zhao, Yi Liu, Yun Wang, Dongning Hao, Jingyun Cen, Shi-Qi Yao, Dan Zhang, Lifang Liu, David Chien Lye, Zhifeng Hao, Tien Yin Wong, Ling-Ping Cen

**Affiliations:** ^1^Joint Shantou International Eye Center of Shantou University and the Chinese University of Hong Kong, Shantou, China; ^2^Shantou University Medical College, Shantou, China; ^3^School of Optometry, The Hong Kong Polytechnic University, Kowloon, Hong Kong SAR, China; ^4^Network & Information Centre, Shantou University, Shantou, China; ^5^Queensland University of Technology, Brisbane, QLD, Australia; ^6^The First Affiliated Hospital of Shantou University Medical College, Shantou, China; ^7^Shantou Healthcare Security Administration Center, Shantou, China; ^8^Hybribio Medical Laboratory Group Ltd., Chaozhou, China; ^9^Human Papillomavirus Molecular Diagnostic Engineering Technology Research Centre, Chaozhou, China; ^10^Yulin First Hospital, Yulin, China; ^11^Beijing Ophthalmology and Visual Sciences Key Laboratory, Beijing Institute of Ophthalmology, Beijing Tongren Hospital, Capital Medical University, Beijing, China; ^12^Jinping District People's Hospital of Shantou, Shantou, China; ^13^Zhengzhou Second Hospital, Henan, China; ^14^Shaoguan University Medical College, Shaoguan, China; ^15^National Centre for Infectious Diseases, Singapore, Singapore; ^16^Tan Tock Seng Hospital, Singapore, Singapore; ^17^Lee Kong Chian School of Medicine, Nanyang Technological University, Singapore, Singapore; ^18^Yong Loo Lin School of Medicine, National University of Singapore, Singapore, Singapore; ^19^College of Mathematics and Computer Science, Shantou University, Shantou, China; ^20^Tsinghua Medicine, Tsinghua University, Beijing, China; ^21^Singapore Eye Research Institute, Singapore National Eye Center, Singapore, Singapore

**Keywords:** COVID-19, zero-COVID policy, Baidu search index, Granger causality test, Bayesian structural time series

## Abstract

**Background:**

China exited strict Zero-COVID policy with a surge in Omicron variant infections in December 2022. Given China’s pandemic policy and population immunity, employing Baidu Index (BDI) to analyze the evolving disease landscape and estimate the nationwide pneumonia hospitalizations in the post Zero COVID period, validated by hospital data, holds informative potential for future outbreaks.

**Methods:**

Retrospective observational analyses were conducted at the conclusion of the Zero-COVID policy, integrating internet search data alongside offline records. Methodologies employed were multidimensional, encompassing lagged Spearman correlation analysis, growth rate assessments, independent sample T-tests, Granger causality examinations, and Bayesian structural time series (BSTS) models for comprehensive data scrutiny.

**Results:**

Various diseases exhibited a notable upsurge in the BDI after the policy change, consistent with the broader trajectory of the COVID-19 pandemic. Robust connections emerged between COVID-19 and diverse health conditions, predominantly impacting the respiratory, circulatory, ophthalmological, and neurological domains. Notably, 34 diseases displayed a relatively high correlation (r > 0.5) with COVID-19. Among these, 12 exhibited a growth rate exceeding 50% post-policy transition, with myocarditis escalating by 1,708% and pneumonia by 1,332%. In these 34 diseases, causal relationships have been confirmed for 23 of them, while 28 garnered validation from hospital-based evidence. Notably, 19 diseases obtained concurrent validation from both Granger causality and hospital-based data. Finally, the BSTS models approximated approximately 4,332,655 inpatients diagnosed with pneumonia nationwide during the 2 months subsequent to the policy relaxation.

**Conclusion:**

This investigation elucidated substantial associations between COVID-19 and respiratory, circulatory, ophthalmological, and neurological disorders. The outcomes from comprehensive multi-dimensional cross-over studies notably augmented the robustness of our comprehension of COVID-19’s disease spectrum, advocating for the prospective utility of internet-derived data. Our research highlights the potential of Internet behavior in predicting pandemic-related syndromes, emphasizing its importance for public health strategies, resource allocation, and preparedness for future outbreaks.

## 1 Introduction

The global COVID-19 pandemic has posed an unprecedented challenge, registering over 700 million confirmed cases and an estimated 7 million fatalities globally by July 2023 (1). Conversely, as of December 23, 2022, China had reported 397,195 confirmed cases and 5,241 deaths (2). Employing stringent quarantine measures under the “Zero-COVID Strategy” from 2020 to late 2022 served as a pivotal approach in curtailing viral transmission and preserving the healthcare infrastructure in China (3). However, the end of this strategy on December 7, 2022, precipitated a significant upsurge in Omicron variant prevalence, a dominant strain in China. China’s distinctive demographic dynamics and policy framework present unique epidemiological complexities. As the most populous nation, it grapples with an aging populace, surpassing 267.36 million individuals aged 60 and above in 2019, where 75% endure chronic conditions like cardiovascular diseases, diabetes, and hypertension, posing intricate public health challenges. Remarkably, China has achieved an impressive vaccination coverage of 90.47%, with 88.01% completing the primary vaccination regimen and 47.61% receiving booster doses (4). Prior investigations in China primarily concentrated on clinical presentations among infected individuals within specific locales and medical facilities. However, a holistic comprehension of the diverse disease patterns emerging from theOmicron-COVID-19 surge subsequent to the Zero-COVID Policy remains elusive.

To address this disparity, we employed Baidu, China’s predominant search engine, commanding a market share of 78.4% as of December 2021 (5). Leveraging disease-specific keywords from the Baidu Index (BDI) for a comprehensive nationwide evaluation. Our exhaustive multidimensional scrutiny, corroborated through Granger causality examinations and hospital-derived data, endeavors to shed light on the evolving landscape of COVID-19 ailments post the Zero-COVID Policy. This comprehensive elucidation endeavors to guide pandemic associated public health strategies and resource allocation.

## 2 Methods

### 2.1 Study design

Utilizing BDI, a comprehensive search for diseases was conducted. Lagged Spearman correlation analysis between “COVID-19 (Xin guan)” and other diseases was employed to investigate which diseases were likely to be secondary to COVID-19 infection. Subsequently, we calculated the growth rate of diseases. If the *p*-value of the growth rate was less than 0.05, the peak of disease search data after the quarantine policy change was considered significant compared with the entire year.

**Figure 1:**
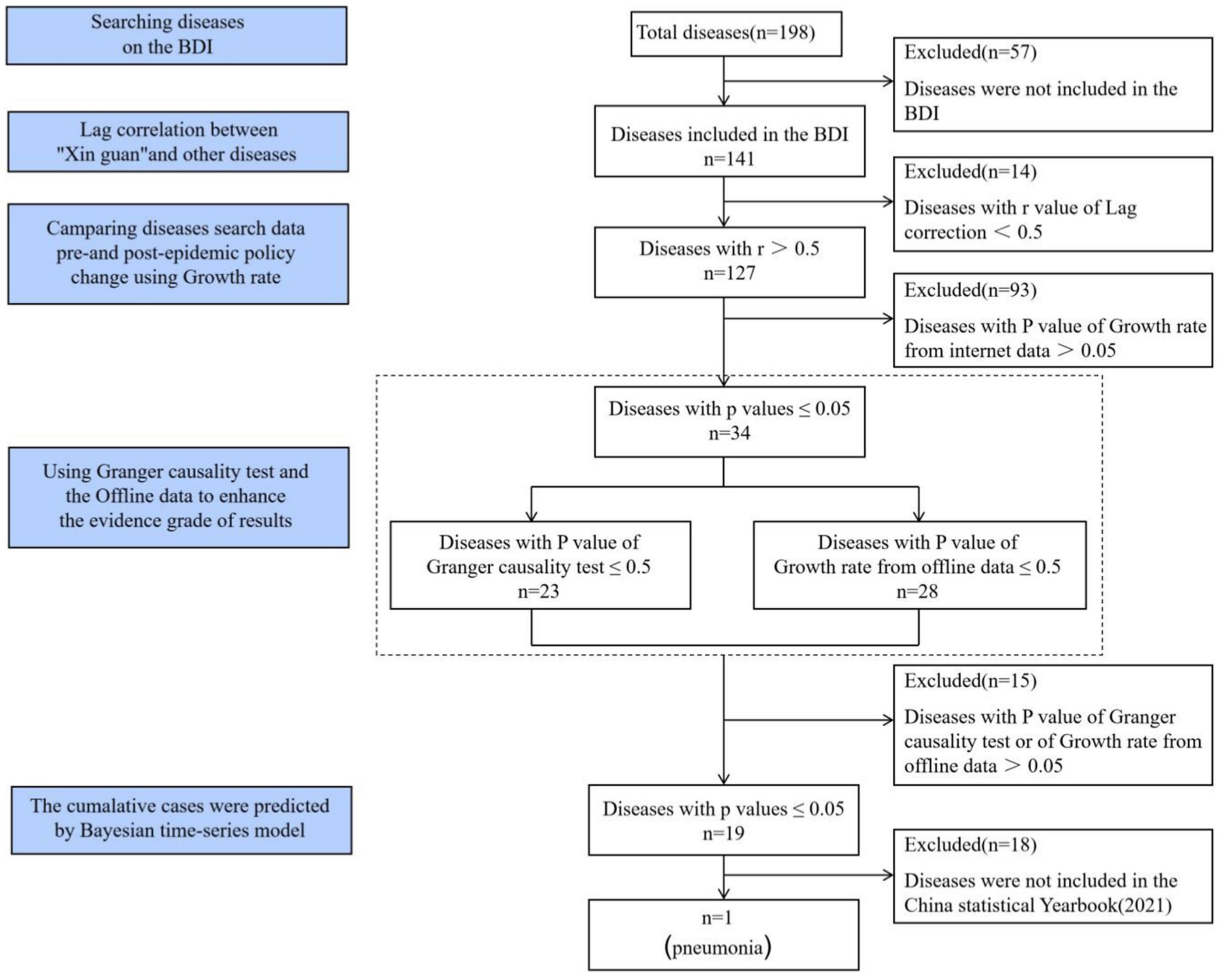
Schematic diagram of the methodology.

Then Granger causality examinations and offline data were used to enhance the evidence grade of diseases with r > 0.5, because that the results derived solely from the internet, which were influenced by various factors, may not comprehensively represent the true scenario. Last, BSTS were deployed to predict the cumulative number of pneumonia inpatients nationwide within the 2 months following the policy change, because “Pneumonia” was included in the Chinese Statistics Yearbook (2021) (6). No patients or the public participation in this study (Figure 1).

### 2.2 Data collection

#### 2.2.1 Internet data

We derived search data from BDI covering the period from January 1, 2021, to June 30, 2023, using a “PC + Mobile” type scope.

#### 2.2.2 Offline data

##### 2.2.2.1 COVID-19 nucleic acid reverse transcription-polymerase chain reaction (RT-PCR) test data

The data were obtained from two facilities, representative of both southern and northern regions in China from January 1, 2021, to June 30, 2023.

##### 2.2.2.2 Hospital-confirmed diseases data

The data were collated from diverse hospitals across six regions in China, covering both outpatient and inpatient data from January 1, 2021, to June 30, 2023.

### 2.3 Statistical analysis

#### 2.3.1 Internet data addition (reducing information bias)

We aggregated search indices of standardized and colloquial expressions for each disease in the BDI to maximize the integrity of the search and obtain a comprehensive dataset. Studying all datasets for each disease as time series and visualizing their search curves using time series plots.

#### 2.3.2 Lagged spearman correlation analysis of internet data (correlations between “COVID-19” and other diseases)

The time series plot revealed a significant peak in the nationwide search curve for “COVID-19” after the lifting of quarantine policies, with the intersection of the peak and the annual mean value line occurring on November 13, 2022, and January 7, 2023. These two intersections represented the start and end dates of the search peak for “COVID-19,” totaling 56 days. Lagged correlation analysis was performed between “COVID-19” and other diseases, grouping data in 56-day intervals. Each disease group commenced on November 13, 2022, shifting daily for maximally statistically significant r (*p* < 0.05, two-tailed).

#### 2.3.3 Growth rate of diseases from internet data (degree of increase in diseases search data)

The maximum value for each disease occurred between December 7, 2022, and February 7, 2023. We calculated the average of the 15 days before and after these values, defining it as the monthly average covering a total of 31 days. Equation 1 is then used to calculate the growth rate.

(1) $$Growthrate=\frac{Monthlyaverage-Annualaverage}{Annualaverage}\times 100\%$$

Monthly average, centered around max value; Annual average, the mean value of the whole year preceding December 7th 2022.

The independent sample T-test was used to compare the monthly average and the annual average (*p* < 0.05, single-tailed).

#### 2.3.4 Granger causality examinations of internet data (enhancing the evidence grade of results)

The time series for “COVID-19” and other diseases were selected from the 4 months before and 2 months after the lifting of quarantine policies (August 10, 2022, to January 4, 2023), totaling 180 days. The Augmented Dickey-Fuller test (ADF test) was used to test the stationarity of time series. This study employed ARIMA models with a lagged parameter (p) greater than 0 for Granger causality examinations, with a *p*-value <0.05.

#### 2.3.5 Growth rate assessments of diseases from offline data (enhancing the evidence grade of results)

Two peaks of concentrated COVID-19 infections occurred in China after the quarantine policy change, within 2 months after the policy change (COVID-19 I wave) and from April to June 2023 (COVID-19 II wave). Using the same methods as previously described to calculate the growth rate of diseases from offline data for the two periods.

#### 2.3.6 Bayesian structural time-series (BSTS) models (to predict the cumulative number of pneumonia inpatients)

The “CausalImpact” package in RStudio was used to fit the BSTS models by selecting the observed data of “Pneumonia” from January 1, 2021 to December 6, 2022 on a weekly basis, and to predict the counterfactual situation for 2 months after December 7, 2022. Equations 2, 3 were used to calculate the cumulative number of pneumonia inpatients in China within 2 months after the quarantine change, defining it as Δ $Y_{t}$ .

(2) $$\frac{\Delta R_{D-BDI}}{\Delta R_{D-R}}=\frac{\Delta R_{W-BDI}}{\Delta R_{W-R}}$$

(3) $$\Delta Y_{t}=\left(\;\Delta R_{W-R}+1\right)*D*S\;$$

ΔR*_D−BDI_*: Relative increase in Shantou city search data; *ΔR_W−BDI_*: Relative increase in nationwide search data; *ΔR_D−R_*: Relative increase in the number of inpatients at Hospital B; *ΔR_W−R_*: Relative increase in the number of inpatients in China; D: The proportion of cumulative search data for the term “pneumonia” in the BDI from December 7, 2021, to February 4, 2022, compared with the entire year 2021; S: The number of pneumonia inpatients nationwide for the entire year 2021, as reported by the Chinese Statistics Yearbook (2021).

## 3 Results

### 3.1 Longitudinal BDI of “COVID-19” correlates with nucleic acid test of COVID-19

**Figure 2:**
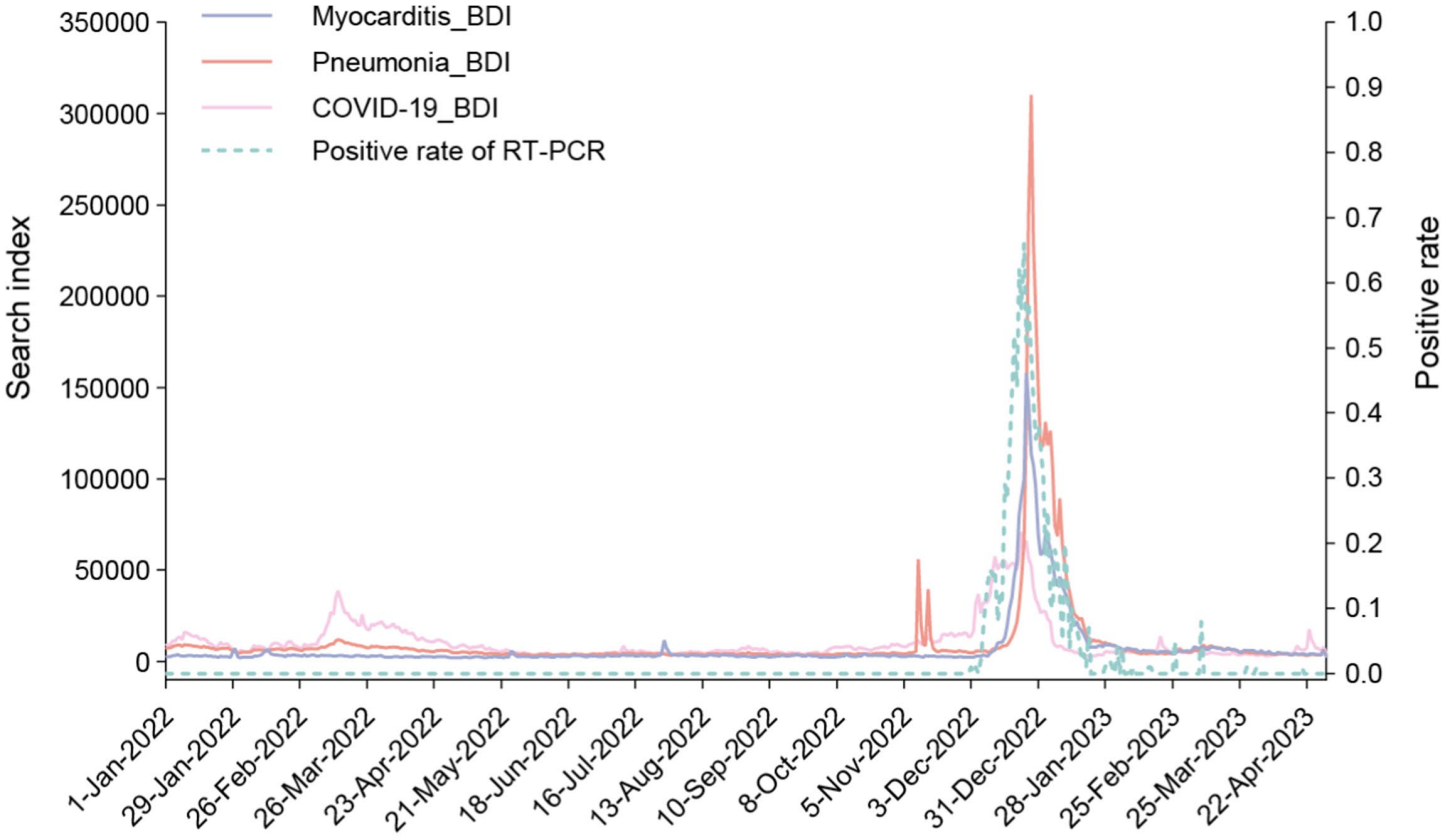
Time series plot of BDI search terms “COVID-19 (Xin guan)”, “Pneumonia” and “Myocarditis,” as well as Nucleic acid positivity rates calculated from RT-PCR test data. This time series graph was drawn with BDI data using the y axis on the left, and positive rate of RT-PCR data using y axis on the right.

A distinct peak was evident in the time series plot of COVID-19, and a comparable pattern was observed in the instances of “Pneumonia” and “Myocarditis,” both recognized as associated with COVID-19 infection (Figure 2). We calculated the RT-PCR test positivity rate from Beijing (with a monthly average testing capacity of 1,243,305 cases) and Shantou (with a monthly average testing capacity of 35,131 cases) (Supplementary Table S1). The positivity rate curve exhibited consistency with the internet data. This reinforced the reliability of utilizing internet data for disease spectrum analysis.

### 3.2 Correlation between “COVID-19” and diseases, as well as the growth rate of diseases from internet data

In this study, searches were conducted for a total of 198 diseases, of which 141 diseases yielded BDI search results. A total of 142 search terms including “COVID-19” and 141 diseases were analyzed (Supplementary Table S2).

**Figure 3:**
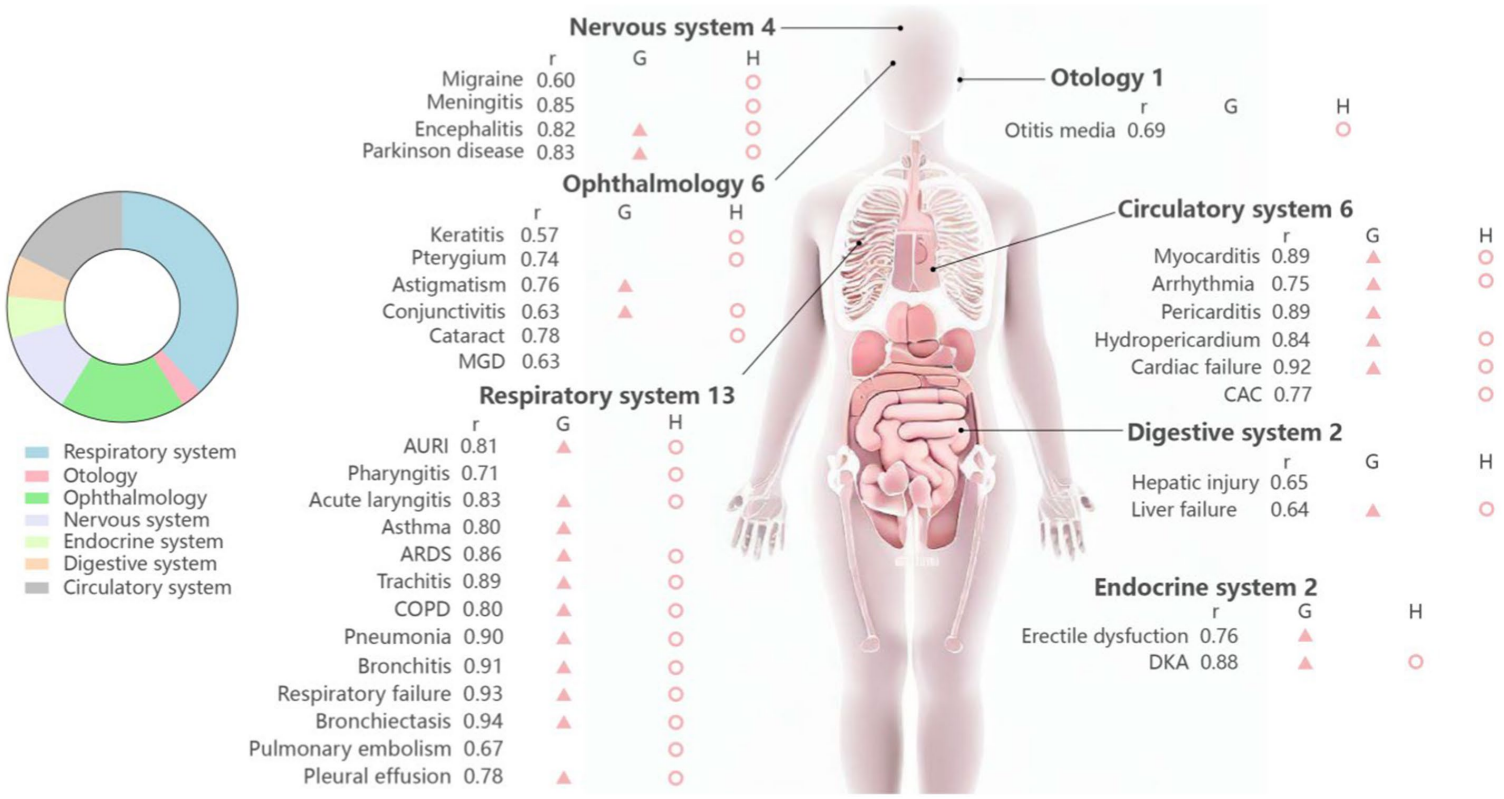
Distribution of positive results from internet data by human body systems in lagged Spearman correlation analysis, growth rate assessments and Granger causality examinations. The red triangle represents the positive results in Granger test of BDI data. The red circle indicates that the disease existed growth rate with statistical significance in analysis of hospital data. “r,” correlation coefficient; “G,” Granger test; “H,” hospital data. MGD, meibomian gland dysfunction; AURI, acute upper respiratory infection; ARDS, acute respiratory distress syndrome; COPD, chronic obstructive pulmonary disease; IPF, idiopathic pulmonary fibrosis; DKA, diabetic ketoacidosis; CAC, coronary atherosclerotic cardiopathy; DN, diabetic nephropathy.

Our analysis showed significant positive correlations between COVID-19 and a broad range of diseases, indicating that during the outbreak of the COVID-19 pandemic, the search index related to specific diseases tended to increase. Among the 141 diseases, 127 exhibited r greater than 0.5, and 16 diseases exhibited r exceeding 0.8, signifying a strong degree of association, with all associated *p*-values falling below the significance threshold of 0.05. Specifically, the top three diseases with the highest r were “Bronchiectasis” (r = 0.94), “Respiratory failure” (r = 0.93), and “Heart failure” (r = 0.92) (Figure 3).

As for the growth rate assessments of diseases from internet data, only 34 diseases passed the independent sample T-tests, including “Pneumonia,” “Myocarditis,” “Meibomian gland dysfunction (MGD) “, “Otitis media,” etc. (Figure 3). These diseases were distributed across systems: respiratory (13/34), circulatory (6/34), ophthalmic (6/34), neurological (4/34), gastrointestinal (2/34), endocrine (2/34), and otologic (1/34). The results show 12 diseases demonstrating growth rates exceeding 50%, including “Myocarditis,” “Pneumonia,” “Meibomian gland dysfunction,” “Acute upper respiratory infection,” “Bronchitis,” “Acute laryngitis,” “Chronic obstructive pulmonary disease,” “Tracheitis,” “Pericarditis,” “Acute respiratory distress syndrome”, “Hydropericardium,” and “Meningitis.” Two diseases exhibited the highest growth rate: “Myocarditis” at 1,708% and “Pneumonia” at 1,332%. This suggests that diseases secondary to COVID-19 were mainly concentrated in the respiratory and circulatory systems.

### 3.3 Granger causal test of “COVID-19” and diseases from internet data

The causality test results indicated statistically significant causal relationships among 23 out of the 34 diseases tested (Figure 3), showing high consistency (23/34), which prove most diseases with r > 0.5 secondary to COVID-19 infection.

The pulmonary embolism did not pass the ADF test, indicating potential non-stationarity in the time series of this disease (Table 1). The *F*-value was used to measure the strongest of causality. Notably, the top three diseases with the strength causal relationship were: “Pneumonia” (*F* = 18.00), “Acute upper respiratory tract infection” (*F* = 16.77), “Pleural effusion” (*F* = 12.02).

**Table 1:** Granger causality test of diseases from internet data.

Diseases	ADF-test *p*-values	Best fitted model	AIC	RMSE	F	Granger causality *p*-values
Asthma	0.66	ARIMA(1,0,0)	2463.15	221.76	2.98	0.0328
Parkinson disease	0.80	ARIMA(1,0,0)	2185.44	102.78	3.06	0.0296
Hepatic injury	0.16	ARIMA(3,0,2)	1890.80	44.16	3.07	0.0294
Pericarditis	0.31	ARIMA(1,0,3)	1923.02	48.59	3.46	0.0176
Cardiac failure	0.62	ARIMA(1,0,2)	2380.52	174.15	5.08	0.0022
Bronchitis	0.80	ARIMA(2,0,2)	2675.96	391.00	6.46	0.0004
Arrhythmia	0.42	ARIMA(1,0,1)	2233.04	116.49	6.51	0.0003
Conjunctivitis	0.55	ARIMA(1,0,0)	2592.13	316.98	6.76	0.0002
Respiratory failure	0.67	ARIMA(1,0,2)	2209.78	108.43	7.26	0.0001
Trachitis	0.82	ARIMA(1,0,1)	2035.43	66.80	7.97	0.0001
Chronic obstructive pulmonary disease	0.15	ARIMA(2,0,1)	2878.29	696.09	8.39	<0.0001
Diabetic ketoacidosis	0.63	ARIMA(3,0,2)	2300.13	137.32	8.94	<0.0001
Bronchiectasis	0.76	ARIMA(1,0,0)	2120.58	85.49	9.09	<0.0001
Acute laryngitis	0.46	ARIMA(2,0,1)	2475.64	225.98	10.33	<0.0001
Hydropericardium	0.71	ARIMA(3,0,0)	2192.64	103.25	10.88	<0.0001
Acute respiratory distress syndrome	0.85	ARIMA(1,0,0)	2011.78	63.25	11.14	<0.0001
Myocarditis	0.36	ARIMA(3,0,3)	3618.55	5314.31	11.56	<0.0001
Pleural effusion	0.55	ARIMA(1,0,0)	2091.29	79.07	12.02	<0.0001
Acute upper respiratory infection	0.28	ARIMA(2,0,1)	2891.57	715.83	16.77	<0.0001
Pneumonia	0.37	ARIMA(2,0,2)	3945.46	13410.76	18.00	<0.0001
Asynodia	0.30	ARIMA(1,0,1)	2722.48	453.68	2.62	0.0528
Coronary atherosclerotic cardiopathy	0.38	ARIMA(2,0,3)	3080.77	1203.89	2.29	0.0797
Astigmatism	0.53	ARIMA(3,0,4)	2388.33	172.47	2.14	0.0965
Otitis media	0.54	ARIMA(1,0,1)	2506.88	248.23	2.01	0.1140
Liver failure	0.12	ARIMA(1,0,0)	2076.66	75.88	2.01	0.1149
Migraine	0.81	ARIMA(1,0,0)	2551.88	283.31	1.67	0.1752
Meibomian gland dysfunction	0.61	ARIMA(2,0,1)	1787.05	33.42	1.42	0.2378
Cataract	0.97	ARIMA(1,0,0)	2365.80	169.03	1.30	0.2776
Pterygium	0.93	ARIMA(1,0,2)	2061.66	71.89	1.03	0.3824
Encephalitis	0.25	ARIMA(1,0,2)	2233.87	115.94	1.00	0.3940
Pharyngitis	0.56	ARIMA(2,0,1)	2632.79	349.38	0.98	0.4015
Keratitis	0.32	ARIMA(1,0,1)	2520.56	259.28	0.61	0.6102
Meningitis	0.52	ARIMA(1,0,0)	2371.58	171.83	0.31	0.8161
Pulmonary embolism	0.02					

### 3.4 Growth rate of diseases from offline data

We gathered outpatient and inpatient data from a cohort of seven hospitals across diverse regions in China, as detailed in Table 2.

**Table 2:** Overview of offline data from various hospitals and Healthcare Security Administration of Shantou.

Organization	Locate	City level	City population (thousands of people)	Cases (person-time)
Hospital A	Shantou City, Guangdong Province (South China)	Prefecture-level city	554.2	2,228,440
Hospital B	Shantou City, Guangdong Province (South China)	District/County	77.7	Inpatient: 19,448 Outpatient: 87,613
Hospital C	Jining City, Shandong Province (East China)	District/County	115.2	Inpatient: 548,336
Hospital D	Yulin City, Shanxi Province (Northwest China)	Prefecture-level city	361.6	Inpatient: 539,129 Outpatient: 611,516
Hospital E	Zhengzhou City, Henan Province (Central China)	Prefecture-level city	1282.8	Inpatient: 654,215 Outpatient: 1,899,789
Hospital F	Enping City, Jiangmen City, Guangdong Province (South China)	County-level city	48.4	Inpatient: 826,702
Hospital G	Huining County, Baiyin City, Gansu Province (Northwest China)	District/County	58	Inpatient: 45,703 Outpatient: 62,877
Sum			2420.2	7,638,165

#### 3.4.1 COVID-19 wave I

The results from offline data analysis confirmed that 28 diseases showed meaningful growth during the first wave of COVID-19, which was highly consistent with the results from internet data (28/34) (Supplementary Table S5). The 28 diseases were similarly concentrated in the respiratory and circulatory systems. The other 6 diseases—“Meibomian gland dysfunction,” “Astigmatism,” “Asthma,” “Pericarditis,” “Liver injury,” and “Erectile dysfunction”—lacked positive findings from offline data.

#### 3.4.2 COVID-19 wave II

**Figure 4:**
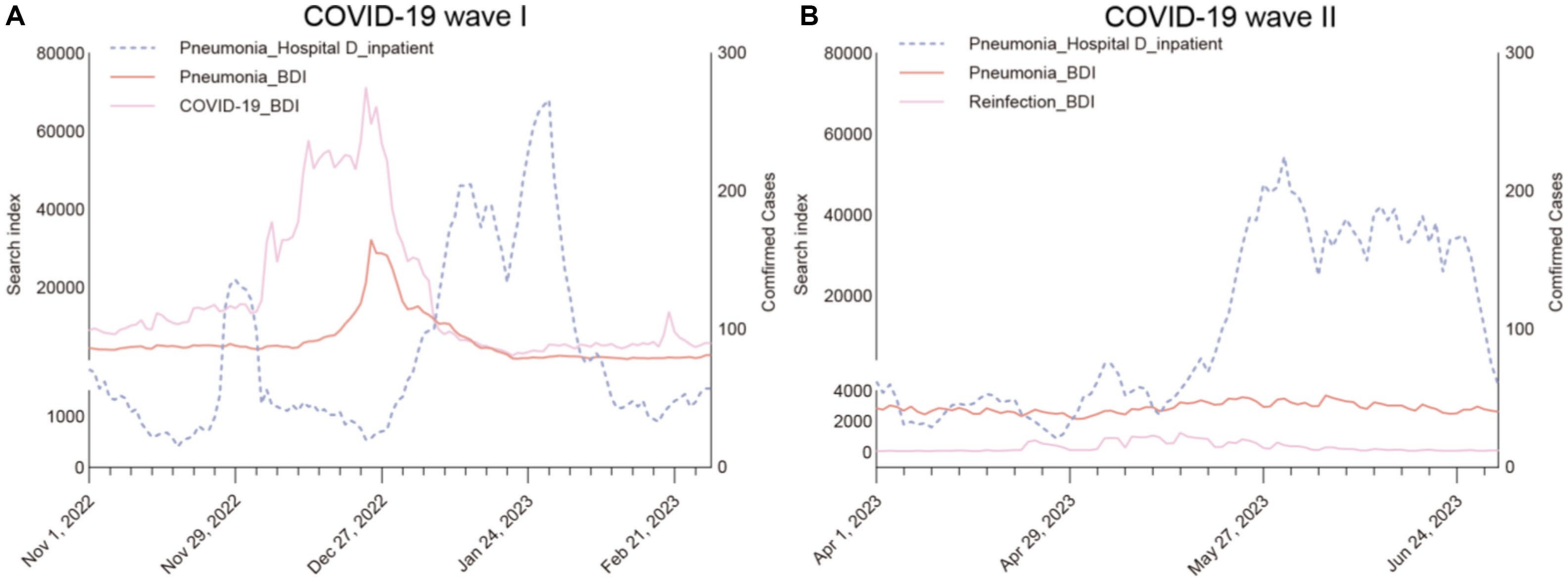
Time series plot of “Pneumonia” from internet data and Hospital D inpatient data. (This time series plot was drawn with BDI data using the y axis on the left, and hospital data using y axis on the right.) **(A)** Displays three time series plot for pneumonia inpatient data of Hospital D, “Pneumonia” and “COVID-19 (Xin guan)” data in the BDI during the first peak period (November 2022 to February 2023). In **(B)**, displays three time series plot for pneumonia inpatient data of Hospital D, “Pneumonia” and “Reinfection (Er yang)” data in the BDI during the second peak period (April 2023 to June 2023).

The 28 diseases also showed a meaningful rise during the second wave across various hospitals (Figure 4). Longitudinally, the growth rate of most diseases during the second wave was lower than in the first wave, indicating a potential decline in disease incidence. However, arrhythmia and COPD stood out with a higher growth rate during this period. When comparing internet searches and actual disease cases between the two waves, both showed distinct peaks during the first wave. However, there was a mild increase in actual cases during the second wave, but it was not mirrored in internet searches.

### 3.5 To predict the cumulative number of pneumonia inpatients

While our investigation utilizing both internet and offline data has elucidated which diseases are impacted by COVID-19 and to what extent, the exact nationwide incidence for specific diseases remains unknown.

According to the Chinese Statistics Yearbook (2021), public hospitals across China admitted a total of 3,251,958 pneumonia patients. Using the BSTS models, we predict that within 2 months after the implementation of unrestricted policies, the cumulative number of hospitalized pneumonia inpatients nationwide reached 4,332,655.

## 4 Discussion

This study amalgamated data from BDI, employing a multi-faceted analysis encompassing lagged Spearman correlation analysis, growth rate assessments, independent sample T-tests, Granger causality, and BSTS models. The research aimed to delineate the disease spectrum of COVID-19 and prognosticate the cumulative count of pneumonia inpatients in China post the end of Zero-COVID Policy, substantiated by hospital-derived data. Adopting a macroscopic viewpoint across the national populace, our study offers insights into the far-reaching impact of COVID-19 on various diseases. This broader perspective contributes significantly to understanding the intricacies of pandemic dynamics, potentially aiding in the judicious allocation of healthcare resources for effective mitigation efforts.

The comprehensive analysis of 34 diseases linked to COVID-19 reveals a predilection for impacts within the respiratory and circulatory systems. Among these, 15 diseases lacked Granger causality or hospital-based data support, while 19 exhibited substantial and corroborative evidence across multiple fronts—including internet data, causal relationship tests, and offline data validation—signifying a more definitive association with COVID-19 infection. This disease spectrum reaffirms the predominant influence of COVID-19 on the respiratory and circulatory systems, aligning with established research patterns (7–9). The established correlation underscores the importance of recognizing the broader ramifications of the pandemic on systemic health, urging a comprehensive approach to public health considerations.

Our investigation notably identified “Otitis media” within the spectrum of diseases, substantiated by a convergence of internet and offline data. This finding contrasts with prior research indicating a reduced incidence during the pandemic (10, 11). The intricate pathophysiological mechanisms implicated encompass viral infections, immune responses, and inflammatory processes (9, 12). This discrepancy may be attributed to factors such as race, viral strains, and other variables that warranting further in-depth examination.

Employing multidimensional analytical methodologies significantly enhances the integrity and precision of findings. Among the 34 diseases showing moderate to strong correlations (r > 0.5) in lagged Spearman correlation analysis based on BDI, 23 demonstrated correlations substantiated by Granger causality examinations, while 28 revealed correlations supported by offline hospital data. Notably, 19 diseases exhibited concurrent support from both Granger causality examinations and hospital data, signifying a heightened level of evidence reinforcing the association between COVID-19 and these diseases. This convergence of evidence from diverse methodologies bolsters the validity of the observed associations between COVID-19 and the spectrum of illnesses studied, elucidating the intricate network of connections between COVID-19 and various diseases. For instance, analysis of internet data suggested potential associations between diseases like “Pterygium” and “Cataracts” with COVID-19, although causality tests were inconclusive. A thorough review of the respective time series plots indicated search curve trends of initial decline followed by an increase post-policy change, hinting at potential inaccuracies due to rebound medical-seeking behavior (13). Furthermore, the appearance of the search term “MGD” on BDI on October 20, 2022, lacking historical data for pre-pandemic annual averages, resulted in false-positive outcomes.

This discovery emphasizes the necessity for meticulous scrutiny and cautious interpretation of web-based data while examining the disease landscape. It accentuates the pivotal role of cross-referencing with clinical archives. Employing multifaceted analytical approaches becomes imperative to expedite the identification of such misleading outcomes. This comprehensive strategy aids in delineating a more exhaustive portrayal of disease dynamics post-policy modification.

Employing the BSTS model, our projection suggests a nationwide total of 4,332,655 hospitalized pneumonia cases within 2 months following the discontinuation of the Zero-COVID policy. This estimation, exceeding the 2021 Chinese Statistical Yearbook’s recorded pneumonia inpatients by approximately one million, likely presents a conservative figure. Notably, our forecast encapsulates solely hospitalized instances, excluding a multitude of mild pneumonia cases managed in outpatient settings. Additionally, resource limitations during outbreaks may potentially skew the representation of severe pneumonia cases, implying an inherent underestimation in our projected count with the genuine incidence Diverse hospital specialties and competencies serve as magnets for patients inclined toward specific disease profiles, resulting in disparate disease propagation rates. Hospitals dedicated to particular medical realms often observe elevated disease incidences pertinent to their expertise. Thus, amalgamating both the Baidu search engine and hospital-derived data becomes imperative to craft a more encompassing narrative that closely mirrors real-world scenarios.

Throughout the secondary surge of COVID-19, several ailments manifested a subsequent rise in offline data. This latter peak demonstrated a marked reduction compared to its antecedent, potentially attributed to diminished possibilities of reinfection or attenuated symptomatology observed in individuals possessing inherent or hybrid immunity against SARS-CoV-2. Furthermore, the dispersed distribution of cases during the second wave extended its duration and augmented the pinnacle of the surge. A prior investigation (14) projected a surge in mortality, approximating 1.87 million deaths within the initial two months subsequent to the cessation of China’s Zero-COVID Policy. These deaths, prevalent among the older adult and vulnerable cohorts during the initial outbreak, likely contributed to the downturn observed in the secondary peak.

Notably, the absence of the second peak in online data, contrary to offline data, prompts consideration. This divergence potentially signifies reduced public engagement during the secondary surge, indicating the potential suitability of internet-derived data for studying abrupt occurrences.

The study’s reliance on BDI for internet data implies potential limitations. The integration of diverse platforms such as WeChat Index and Weibo Index appears as a promising approach to bolster the robustness of our findings. The hospital data’s focal point within specific Chinese provinces—Shandong, Shaanxi, Gansu, Henan, and Guangdong—poses a restriction. Future investigations should aim for a comprehensive national scope, encompassing data from all provinces. This strategic expansion accounts for regional disparities attributed to geographic, climatic, and socioeconomic variations, crucial for a more accurate depiction of the nationwide disease spectrum.

The present study, however, is subject to certain limitations. There are concerns about sampling bias, because BDI is related to internet access and search behavior. In addition, this study does not encompass all significant diseases. To obtain a more comprehensive understanding of the situation, it is imperative to expand the research scope in future studies.

## 5 Conclusion

Our investigation delved into the impact of COVID-19 on post-Zero-COVID Policy disease patterns. Following the termination of China’s Zero-COVID policy, our study unveiled BDI indicators linking Omicron variant infections to a spectrum encompassing respiratory, circulatory, ophthalmological, and neurological disorders. These findings, backed by Granger causality examinations and hospital data, carry substantial implications. Leveraging the BSTS model, our estimation surpassed 4.3 million nationwide pneumonia inpatients within 2 months of policy relaxation. The potential of search engines in forecasting pandemic-related syndromes offers crucial insights for public health strategies, resource allocation, and future outbreak preparedness.

## Declaration of generative AI and AI-assisted technologies in the writing process

During the preparation of this work the author(s) used ChatGPT 3.5 in order to edit the entire article, correct grammar errors, and make the sentences more coherent and academically styled. After using this tool, the authors reviewed and edited the content as needed and take full responsibility for the content of the publication.

## Data Availability

The original contributions presented in the study are included in the article/Supplementary material, further inquiries can be directed to the corresponding authors.

## Supplementary material

SUPPLEMENTARY TABLE 1 The growth rate of diseases from internet and offline data.
