# Supplementary material for: Multi-dimensional epidemiology and informatics data on COVID-19 wave at the end of zero COVID policy in China
Source: Front Public Health. 2024 Aug 19;12:1442728. doi: 10.3389/fpubh.2024.1442728 (PMC11366567; doi:10.3389/fpubh.2024.1442728)
Supplement: Supplementary file 2 [file Data_Sheet_2.PDF]

Supplementary Material

1. Supplementary methods

1.1. Lag Spearman correlation

Internet searches and social media data have been reported to correlate with traditional surveillance data and can reflect the outbreak of diseases or epidemics in advance[1–3]. Lag correlation was used to find the maximum correlation between COVID-19 and other diseases, because outbreak of the "COVID-19" and various diseases may not occur at the same time. Since the data were not normally distributed, we picked the Spearman correlation instead of Pearson correlation.

1.2. Granger causality tests

Correlation analysis doesn't indicate whether there is a causal relationship between two time series and causal direction[4]. An effective tool to investigate the relationship between "COVID-19" and each disease is the Granger causality tests. Granger causality tests is a time series test based on statistical modeling employed to study causal links among random variables[5]. This method is based on the premise that the cause happens prior to its effect, if factor 1 causes factor 2, then the past values of factor 1 should contain information that helps the prediction of factor 2.

Firstly, the time series of "COVID-19" (x) and various diseases (y) were selected from the six months before and two months after the ending of China's Zero-COVID Policy, a total of 180 days of BDI data.

Secondly, the Augmented Dickey-Fuller test (ADF test) was used to test the stationarity of the time series. H0 indicates the presence of nonstationarity, so rejection of the null hypothesis indicates stationarity of the series. Unstable time series will be discarded, and the time series satisfying the stationarity will be modeled by Auto-Regressive Moving Average （ARIMA） model, which combines the characteristics of moving average and regression methods. Here, the "auto.arima" package in RStudio software is used to automatically select the best modeling parameters. (p, d, q) represents the ARIMA model autoregressive lag order, difference order and moving average lag order[6]. The selection criterion of the best modeling parameter was the minimum AIC(Akaike information criterion) under the premise that the autoregrestic lag term p was not 0.

Finally, Granger causality tests were performed on the "COVID-19" time series and other disease series at the 5% significance level. In this test, H0 means that x is not the cause of y; If p-value<0.05 would reject the null hypothesis, indicating that x is the cause of y, that is, COVID-19 is the cause of the diseases.

1.3. Bayesian structural time-series (BSTS) models

Bayes statistical prediction method is a time series prediction method with dynamic model as the research object. Its basic idea is to combine people's experience information into the actual model as known conditions, that is, to use model information, data information and prior information (information about the unknown parameters of the population distribution)[7] to make predictions.

BSTS models consists of three main parts: the kalman filtering (used to estimate the trend of the target sequence and seasonal), Spike - and - slab method (for variable selection) and bayesian model average (for the final forecast), it has been used to infer that the effect of intervention, such as policy, vaccinations, etc. The model can construct a counterfactual based on the idea of combining a set of candidate predictor variables into a single "comprehensive control"[8,9].

Therefore, based on the idea of counterfactual and comprehensive control, we used the BSTS models to predict the accumulative number of pneumonia inpatients nationwide within the two months following the policy change.

2. Reference

1. Shin SY, Seo DW, An J, et al. High correlation of Middle East respiratory syndrome spread with Google search and Twitter trends in Korea. Sci Rep. 2016;6:32920. doi:10.1038/srep32920.
2. Li C, Chen LJ, Chen X, Zhang M, Pang CP, Chen H. Retrospective analysis of the possibility of predicting the COVID-19 outbreak from Internet searches and social media data, China, 2020. Euro Surveill. 2020;25(10):2000199. doi:10.2807/1560-7917.
3. Santangelo OE, Provenzano S, Piazza D, Giordano D, Calamusa G, Firenze A. Digital epidemiology: assessment of measles infection through Google Trends mechanism in Italy. Ann Ig. 2019;31(4):385-391. doi:10.7416/ai.2019.2300.
4. Lamsal R, Harwood A, Read MR. Twitter conversations predict the daily confirmed COVID-19 cases. Appl Soft Comput. 2022;129:109603. doi:10.1016/j.asoc.2022.109603.
5. Hamta A, Saghafipour A, Zanjirani Farahani L, Moradi Asl E, Ghorbani E. The Granger causality analysis of the impact of climatic factors on visceral leishmaniasis in northwestern Iran in 1995–2019. J Parasit Dis. 2021;45(1):17-23. doi:10.1007/s12639-020-01271-z.
6. Schwarz G. Estimating the Dimension of a Model. The Annals of Statistics. 1978;6(2):461-4, 4.
7. Ding S, Wang J, Ji Y, Hu P. Bayesian statistical analysis of time series prediction models. Public health in China. 2002(09):119-20.
8. Scott SL, Varian HR. Predicting the Present with Bayesian Structural Time Series. SSRN Electronic Journal. 2014;5(1/2):4-23.
9. Xiao J, Zhu Q, Yang F, et al. The impact of enterovirus A71 vaccination program on hand, foot, and mouth disease in Guangdong, China: A longitudinal surveillance study. J Infect. 2022;85(4):428-435. doi:10.1016/j.jinf.2022.06.020.

3. Supplementary Figures

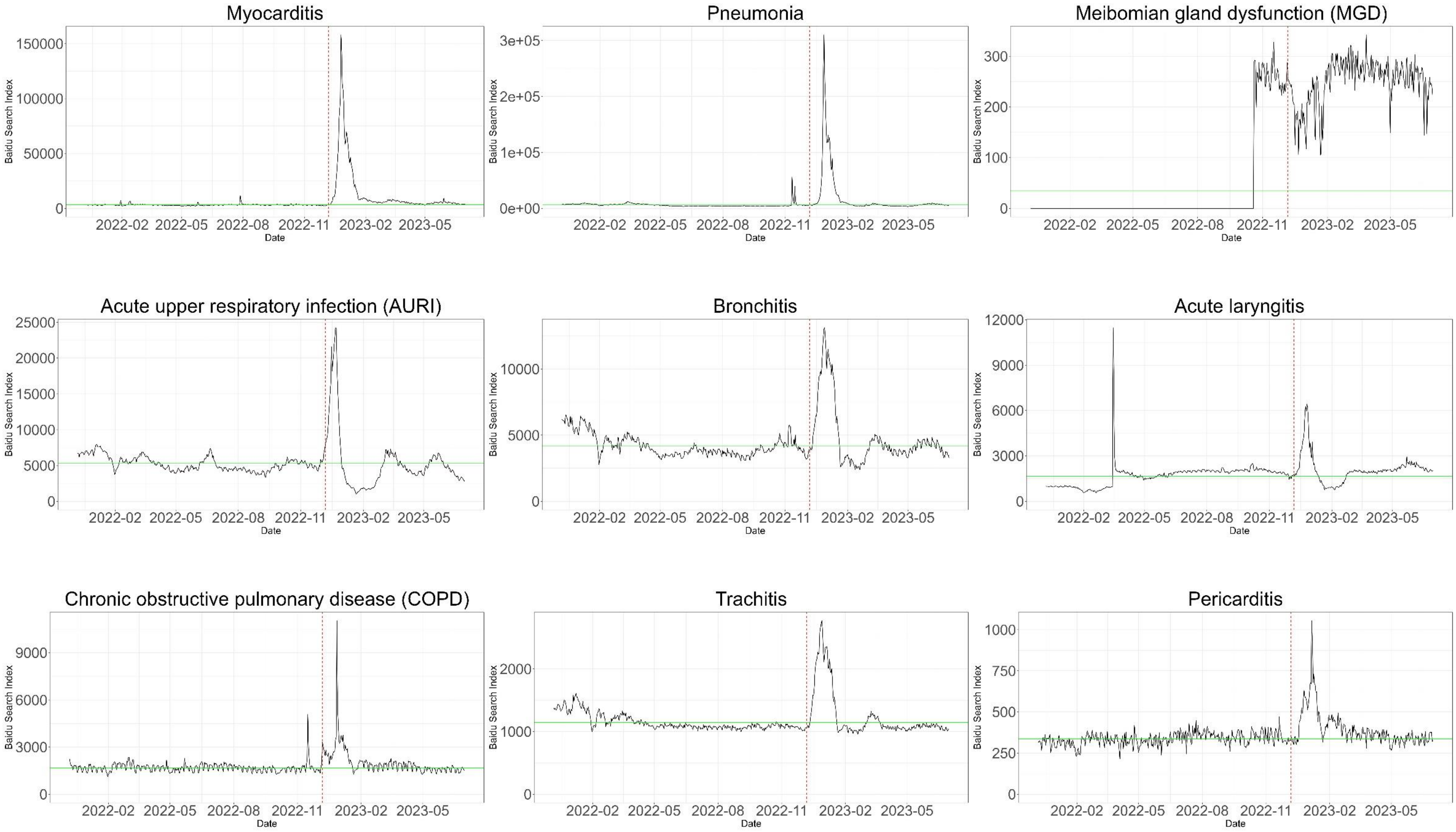

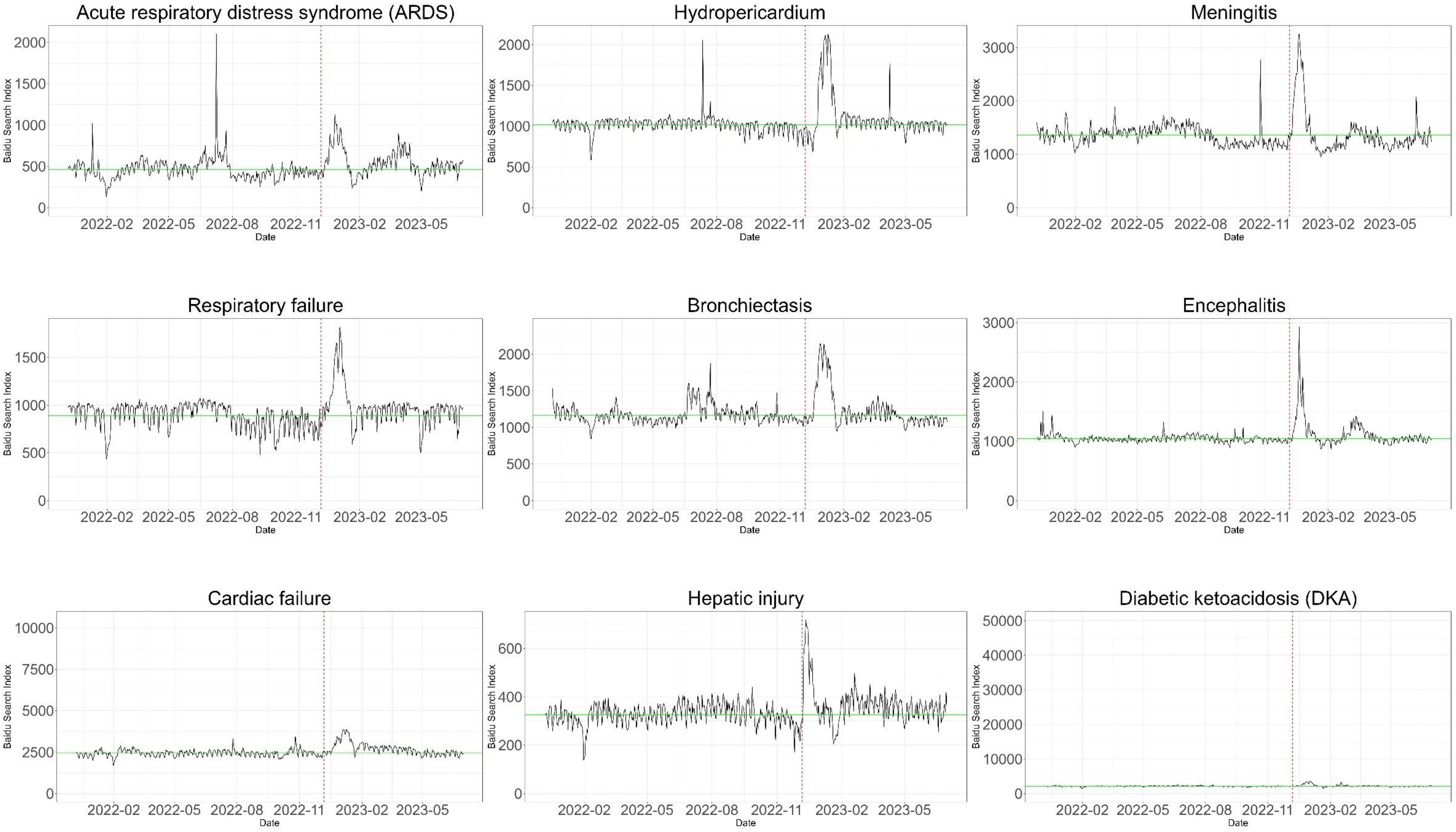

Acute respiratory distress syndrome (ARDS)
Hydropericardium
Meningitis
Respiratory failure
Bronchiectasis
Encephalitis
Cardiac failure
Hepatic injury
Diabetic ketoacidosis (DKA)
Baidu Search Index
Date
2022-02
2022-05
2022-08
2022-11
2023-02
2023-05
0
500
1000
1500
2000
3000
2500
5000
7500
10000
200
400
600
10000
20000
30000
40000
50000

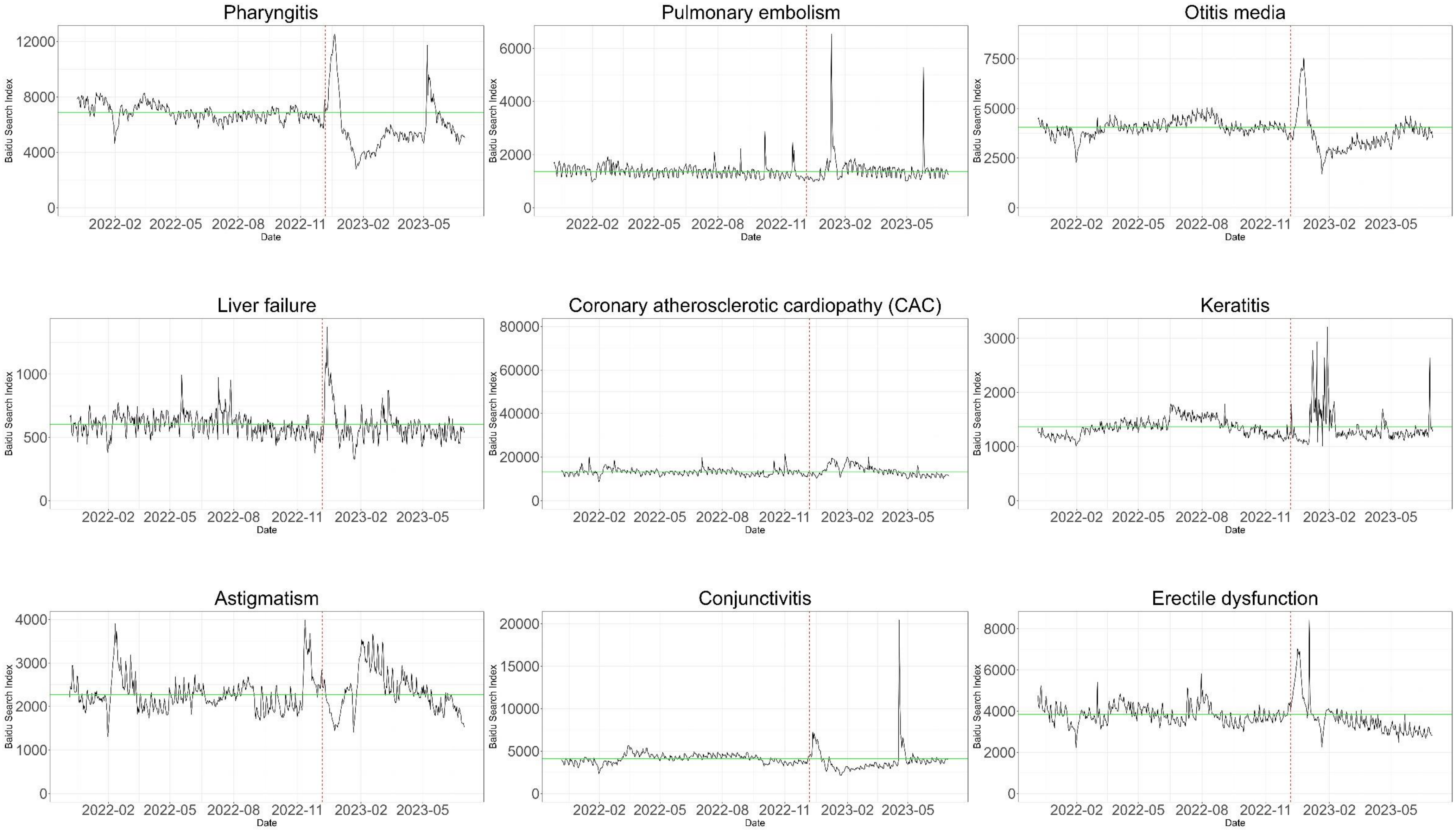

Pharyngitis
Baidu Search Index
12000
8000
4000
0
2022-02
2022-05
2022-08
2022-11
2023-02
2023-05
Date
Pulmonary embolism
Baidu Search Index
6000
4000
2000
0
2022-02
2022-05
2022-08
2022-11
2023-02
2023-05
Date
Otitis media
Baidu Search Index
7500
5000
2500
0
2022-02
2022-05
2022-08
2022-11
2023-02
2023-05
Date
Liver failure
Baidu Search Index
1000
500
0
2022-02
2022-05
2022-08
2022-11
2023-02
2023-05
Date
Coronary atherosclerotic cardiopathy (CAC)
Baidu Search Index
80000
60000
40000
20000
0
2022-02
2022-05
2022-08
2022-11
2023-02
2023-05
Date
Keratitis
Baidu Search Index
3000
2000
1000
0
2022-02
2022-05
2022-08
2022-11
2023-02
2023-05
Date
Astigmatism
Baidu Search Index
4000
3000
2000
1000
0
2022-02
2022-05
2022-08
2022-11
2023-02
2023-05
Date
Conjunctivitis
Baidu Search Index
20000
15000
10000
5000
0
2022-02
2022-05
2022-08
2022-11
2023-02
2023-05
Date
Erectile dysfunction
Baidu Search Index
8000
6000
4000
2000
0
2022-02
2022-05
2022-08
2022-11
2023-02
2023-05
Date

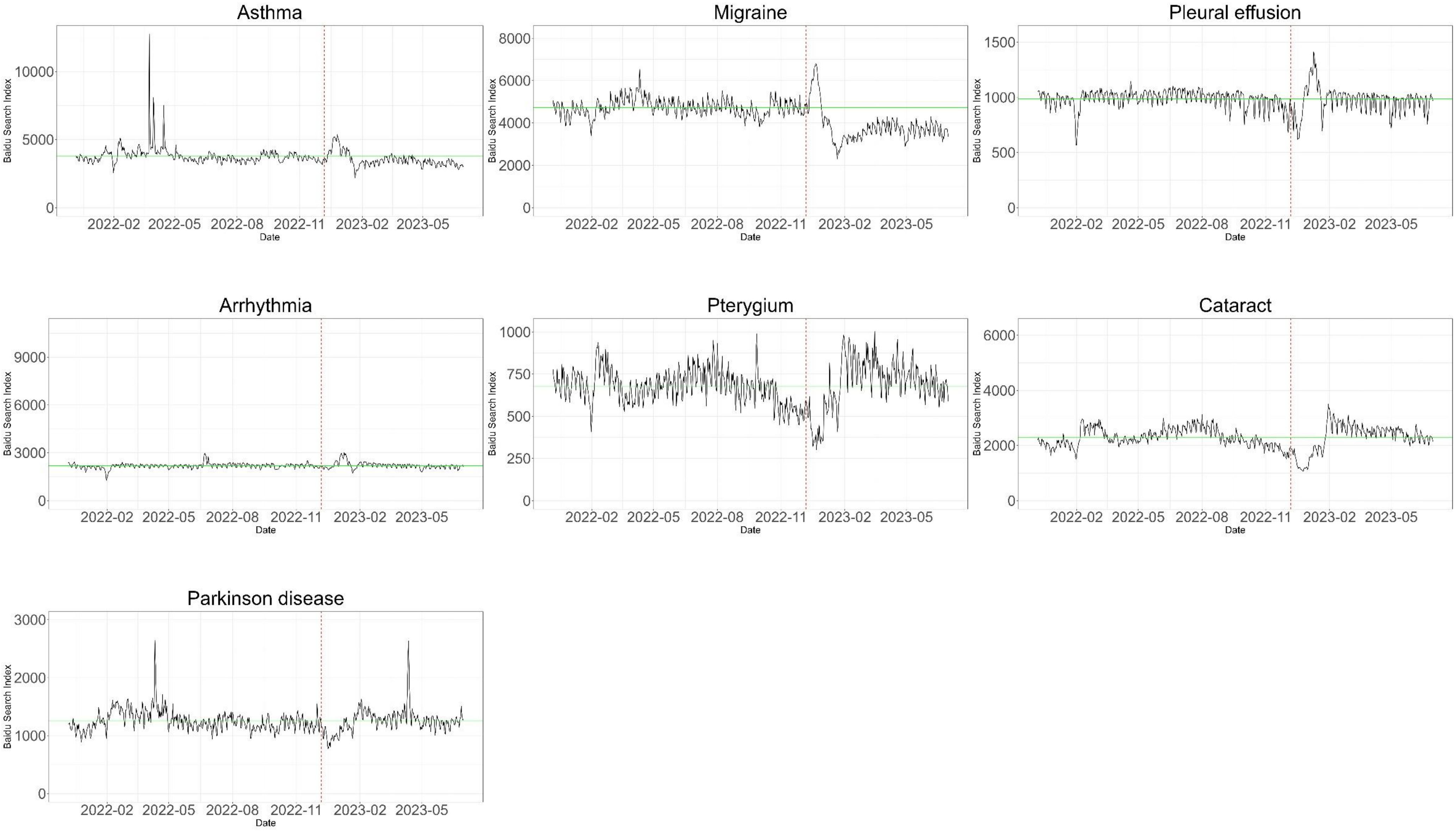

Supplementary figure 1: Time series plot of 35 diseases from BDI which exhibited r greater than 0.5 and passed the t-Tests

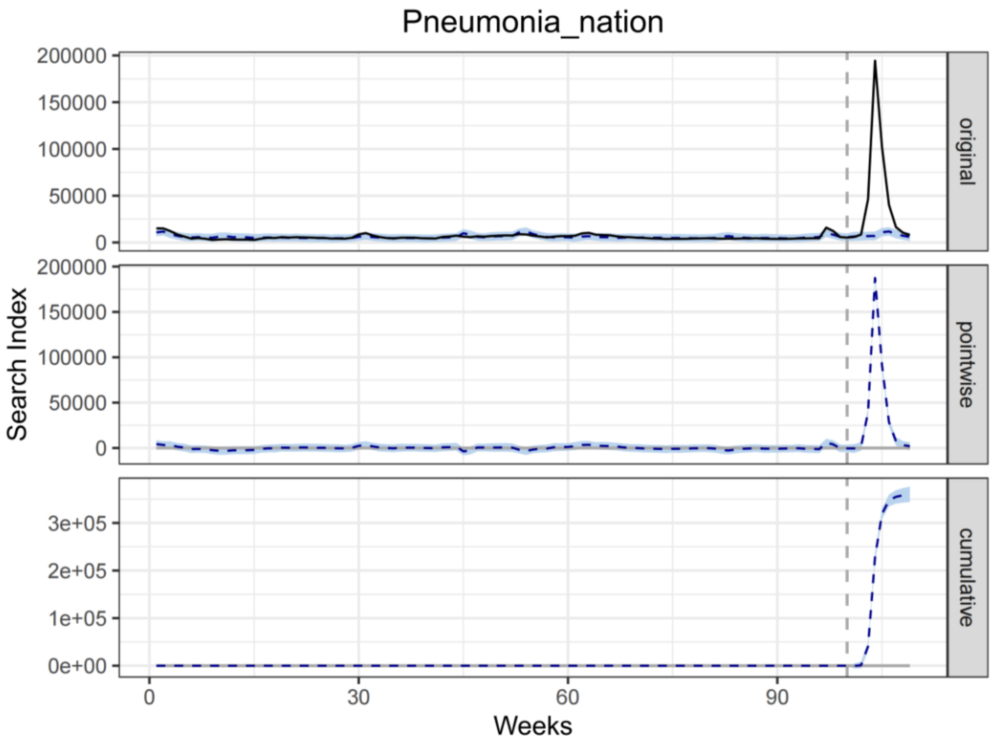

Supplementary figure 2A

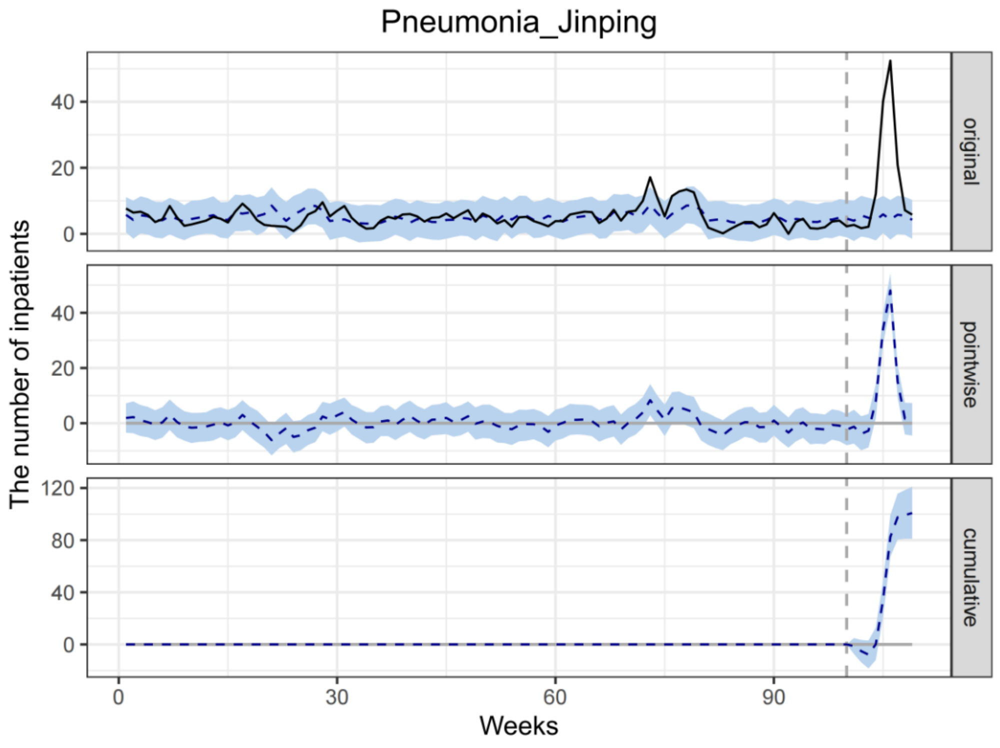

Supplementary figure 2B

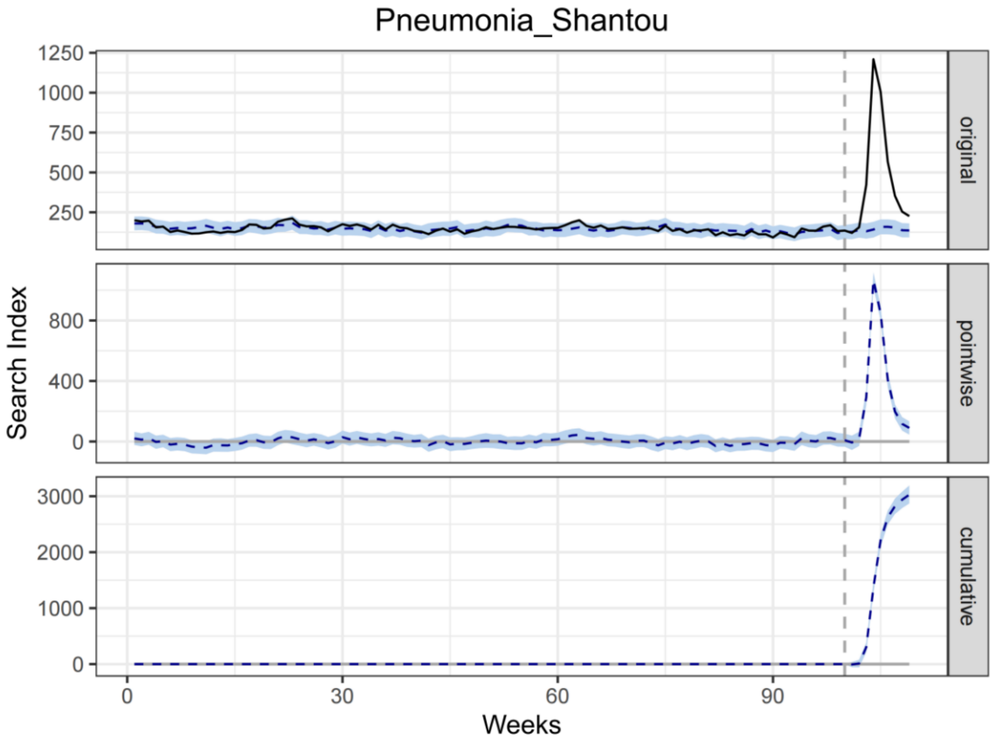

Supplementary figure 2C

Supplementary figure 2: Using the BSTS models to predict the number of inpatients diagnosed with pneumonia nationwide during the two months subsequent to the policy relaxation by through counterfactual (blue shadow: 95% confidence interval for prediction).

Supplementary figure 2A:（original）The dashed line is the nationwide search curve for the keyword "Pneumonia" on the BDI. The solid line is the simulated trajectory of a search curve for the keyword "Pneumonia" with an intervention beginning on December 7, 2022. (pointwise) The dashed line is the difference between the observed data and the counterfactual prediction, the inferred effect of the intervention. (cumulative) The dashed line represents the cumulative amount of effect in the intervention impact.

Supplementary figure 2B:（original）The dashed line is the time series of the number of inpatients at Jinping District People's Hospital. The solid line is the simulated trajectory of a time series of the number of inpatients with an intervention beginning on December 7, 2022. (pointwise) The dashed line is the difference between the observed data and the counterfactual prediction, the inferred effect of the intervention. (cumulative) The dashed line represents the cumulative amount of effect in the intervention impact.

Supplementary figure 2C:（original）The dashed line is the Shantou city-wide search curve for the keyword "Pneumonia" on the BDI. The solid line is the simulated trajectory of a search curve for the keyword "Pneumonia" with an intervention beginning on December 7, 2022. (pointwise) The dashed line is the difference between the observed data and the counterfactual prediction, the inferred effect of the intervention. (cumulative) The dashed line represents the cumulative amount of effect in the intervention impact.
