# Supplementary material for: Multi-dimensional epidemiology and informatics data on COVID-19 wave at the end of zero COVID policy in China
Source: Front Public Health. 2024 Aug 19;12:1442728. doi: 10.3389/fpubh.2024.1442728 (PMC11366567; doi:10.3389/fpubh.2024.1442728)
Supplement: Supplementary file 3 [file Table_1.docx]

| **Diseases** | **r (Online)** | **Growth rate(%)** | | | | | | | | | | | | | | | | | | | | | | | | | | | | | | | | |
| --- | --- | --- | --- | --- | --- | --- | --- | --- | --- | --- | --- | --- | --- | --- | --- | --- | --- | --- | --- | --- | --- | --- | --- | --- | --- | --- | --- | --- | --- | --- | --- | --- | --- | --- |
|  |  | **Online** | **Inpatient** | | | | | | | | | | | | | |  | **Outpatient** | | | | | | | | | | | | | |  | **Hospital A** | |
|  |  |  | **Hospital C** | |  | **Hospital D** | |  | **Hospital B** | |  | **Hospital E** | |  | **Hospital F** | |  | **Hospital G** | |  | **Hospital D** | |  | **Hospital B** | |  | **Hospital E** | |  | **Hospital G** | |  |  |  |
|  |  |  | **Infection** | **Reinfection** |  | **Infection** | **Reinfection** |  | **Infection** | **Reinfection** |  | **Infection** | **Reinfection** |  | **Infection** | **Reinfection** |  | **Infection** | **Reinfection** |  | **Infection** | **Reinfection** |  | **Infection** | **Reinfection** |  | **Infection** | **Reinfection** |  | **Infection** | **Reinfection** |  | **Infection** | **Reinfection** |
| Myocarditis | 0.89^***^ | 1708^***^ | 2255^***^ | 2490^**^ |  | 707^***^ | 152^**^ |  |  |  |  | 410^***^ | 236^***^ |  | 747^**^ |  |  |  |  |  | 1096^***^ |  |  |  |  |  | 349^**^ |  |  |  |  |  | 85^***^ | 38^**^ |
| Pneumonia | 0.9^***^ | 1332^***^ | 34^***^ | 19^***^ |  | 143^***^ | 149^***^ |  | 473^***^ | 41^***^ |  | 294^***^ | 182^***^ |  | 197^***^ |  |  | 1235^***^ |  |  | 144^***^ | 227^***^ |  | 773^***^ | 94^**^ |  | 167^***^ |  |  | 1882^***^ | 253^**^ |  | 44^***^ |  |
| Meibomian gland dysfunction | 0.63^***^ | 612^***^ |  |  |  |  |  |  |  |  |  |  |  |  |  |  |  |  |  |  |  |  |  |  |  |  |  |  |  |  |  |  |  |  |
| Acute upper respiratory infection | 0.81^***^ | 137^***^ | 146^***^ |  |  |  |  |  |  |  |  |  |  |  | 641^***^ |  |  |  |  |  |  |  |  | 171^***^ | 47^***^ |  | 169^***^ | 52^***^ |  |  |  |  | 87^***^ | 177^***^ |
| Bronchitis | 0.91^***^ | 132^***^ | 23^***^ |  |  |  |  |  |  |  |  |  |  |  |  |  |  | 141^***^ | 176^***^ |  | 118^***^ | 133^***^ |  | 146^***^ | 34^**^ |  | 93^***^ | 56^***^ |  | 258^***^ | 439^***^ |  |  |  |
| Acute laryngitis | 0.83^***^ | 111^***^ |  |  |  |  |  |  |  |  |  |  |  |  |  |  |  |  |  |  |  |  |  |  |  |  | 89^*^ | 159^***^ |  | 724^*^ |  |  | 123^***^ | 77^***^ |
| Chronic obstructive pulmonary disease | 0.8^***^ | 95^***^ | 29^***^ | 36^***^ |  | 144^***^ |  |  | 101^***^ | 106^***^ |  | 105^***^ | 93^***^ |  | 125^***^ |  |  | 230^***^ |  |  |  |  |  |  |  |  |  |  |  | 56^**^ |  |  | 31^**^ | 55^***^ |
| Trachitis | 0.89^***^ | 85^***^ | 24^***^ |  |  |  |  |  |  |  |  |  |  |  |  |  |  | 141^***^ | 176^***^ |  | 118^***^ | 131^***^ |  |  |  |  | 91^***^ | 54^***^ |  | 263^***^ | 437^***^ |  |  |  |
| Pericarditis | 0.89^***^ | 74^***^ |  |  |  |  |  |  |  |  |  |  |  |  |  |  |  |  |  |  |  |  |  |  |  |  |  |  |  |  |  |  |  |  |
| Acute respiratory distress syndrome | 0.86^***^ | 67^***^ |  |  |  |  |  |  |  |  |  | 2255^***^ | 1666^***^ |  |  |  |  |  |  |  |  |  |  |  |  |  |  |  |  |  |  |  | 87^***^ |  |
| Hydropericardium | 0.84^***^ | 57^***^ | 2844^*^ |  |  |  |  |  |  |  |  | 113^***^ | 85^***^ |  |  |  |  |  |  |  |  |  |  |  |  |  |  | 264^**^ |  |  |  |  | 31^**^ | 34^**^ |
| Meningitis | 0.85^***^ | 55^***^ | 756^***^ |  |  |  |  |  |  |  |  |  |  |  |  |  |  |  |  |  |  |  |  |  |  |  |  |  |  |  |  |  | 72^***^ | 85^***^ |
| Respiratory failure | 0.93^***^ | 48^***^ | 1773^***^ | 395^***^ |  | 121^***^ |  |  |  |  |  | 529^***^ | 173^***^ |  |  |  |  |  |  |  |  |  |  |  |  |  | 821^**^ |  |  |  |  |  | 48^***^ | 98^***^ |
| Bronchiectasis | 0.94^***^ | 46^***^ |  |  |  |  |  |  | 51^**^ |  |  | 45^***^ | 74^***^ |  | 63^***^ |  |  |  |  |  |  |  |  |  |  |  |  |  |  |  |  |  |  |  |
| Encephalitis | 0.82^***^ | 38^***^ | 285^***^ |  |  |  |  |  |  |  |  | 172^***^ |  |  |  |  |  |  |  |  |  |  |  |  |  |  |  |  |  |  |  |  | 81^***^ | 292^***^ |
| Cardiac failure | 0.92^***^ | 35^***^ |  |  |  | 18^**^ | 58^***^ |  |  |  |  | 140^***^ |  |  | 457^***^ |  |  |  |  |  |  |  |  |  |  |  | 70^***^ |  |  |  |  |  | 14^***^ | 13^***^ |
| Hepatic injury | 0.65^***^ | 33^***^ |  |  |  |  |  |  |  |  |  |  |  |  |  |  |  |  |  |  |  |  |  |  |  |  |  |  |  |  |  |  |  |  |
| Diabetic ketoacidosis | 0.88^***^ | 29^***^ |  |  |  | 92^**^ | 22^**^ |  |  |  |  | 49^***^ |  |  | 30^*^ |  |  |  |  |  |  |  |  |  |  |  | 52^*^ |  |  |  |  |  | 116^***^ | 81^***^ |
| Pharyngitis | 0.71^***^ | 26^***^ |  |  |  |  |  |  |  |  |  |  |  |  |  |  |  |  |  |  |  |  |  | 281^***^ | 201^***^ |  | 76^**^ | 28^**^ |  |  |  |  |  |  |
| Pulmonary embolism | 0.67^***^ | 26^*^ | 18^*^ | 55^**^ |  | 153^***^ | 29^*^ |  |  |  |  |  |  |  |  |  |  |  |  |  |  |  |  |  |  |  |  |  |  |  |  |  |  |  |
| Otitis media | 0.69^***^ | 25^***^ |  |  |  |  |  |  | 489^**^ | 489^**^ |  | 207^***^ | 133^***^ |  |  |  |  |  |  |  | 25^*^ | 85^***^ |  |  |  |  | 90^***^ | 148^***^ |  |  |  |  |  |  |
| Liver failure | 0.64^***^ | 24^**^ | 178^***^ | 390^***^ |  | 120^***^ | 110^***^ |  |  |  |  | 346^***^ |  |  |  |  |  |  |  |  |  |  |  |  |  |  |  |  |  |  |  |  |  |  |
| Coronary atherosclerotic cardiopathy | 0.77^***^ | 24^***^ | 32^***^ | 30^***^ |  |  |  |  |  |  |  | 30^**^ | 54^***^ |  | 133^***^ | 134^***^ |  |  |  |  |  |  |  | 111^**^ | 137^***^ |  | 63^***^ | 60^***^ |  |  |  |  |  |  |
| Keratitis | 0.57^***^ | 24^***^ |  |  |  |  |  |  |  |  |  | 65^**^ | 61^*^ |  | 661^***^ |  |  |  |  |  |  |  |  |  |  |  | 19^*^ | 43^***^ |  |  |  |  |  |  |
| Astigmatism | 0.76^***^ | 23^***^ |  |  |  |  |  |  |  |  |  |  |  |  |  |  |  |  |  |  |  |  |  |  |  |  |  |  |  |  |  |  |  |  |
| Conjunctivitis | 0.63^***^ | 18^***^ |  |  |  |  |  |  |  |  |  |  |  |  |  |  |  |  |  |  |  |  |  |  |  |  |  |  |  | 220^**^ | 105^*^ |  | 96^*^ |  |
| Erectile dysfunction | 0.76^***^ | 17^**^ |  |  |  |  |  |  |  |  |  |  |  |  |  |  |  |  |  |  |  |  |  |  |  |  |  |  |  |  |  |  |  |  |
| Asthma | 0.8^***^ | 17^***^ |  |  |  |  |  |  |  |  |  |  |  |  | 193^**^ |  |  | 306^***^ | 108^*^ |  |  |  |  |  |  |  |  |  |  |  |  |  |  |  |
| Migraine | 0.6^***^ | 12^***^ |  |  |  |  |  |  |  |  |  |  |  |  |  |  |  |  |  |  |  |  |  | 1827^***^ |  |  |  |  |  |  |  |  |  |  |
| Pleural effusion | 0.78^***^ | 12^***^ | 82^***^ | 46^***^ |  | 24^**^ | 57^***^ |  | 56^***^ | 11^*^ |  | 107^***^ | 45^***^ |  |  |  |  |  |  |  |  |  |  |  |  |  |  |  |  |  |  |  | 47^***^ | 16^***^ |
| Arrhythmia | 0.75^***^ | 11^***^ | 18^***^ | 54^***^ |  | 44^***^ | 91^***^ |  |  |  |  | 57^***^ | 92^***^ |  | 172^***^ |  |  | 52^***^ |  |  |  |  |  | 482^***^ | 780^***^ |  |  |  |  |  |  |  |  |  |
| Pterygium | 0.74^***^ | 11^**^ | 14^*^ | 13^**^ |  |  |  |  |  |  |  | 48^*^ | 111^***^ |  |  |  |  |  |  |  |  |  |  |  |  |  | 53^**^ | 56^***^ |  | 317^**^ | 391^**^ |  |  |  |
| Cataract | 0.78^***^ | 10^*^ |  |  |  |  |  |  | 1077^**^ |  |  | 54^**^ | 116^***^ |  |  |  |  |  |  |  | 60^*^ | 56^*^ |  |  |  |  | 62^**^ | 102^***^ |  |  |  |  |  |  |
| Parkinson disease | 0.83^***^ | 8^***^ | 147^**^ |  |  |  |  |  |  |  |  | 148^***^ |  |  |  |  |  |  |  |  |  |  |  |  |  |  |  |  |  |  |  |  |  |  |
